# Metabolic role of pyrophosphate-linked phosphofructokinase *pfk* for C1 assimilation in *Methylotuvimicrobium alcaliphilum* 20Z

**DOI:** 10.1186/s12934-020-01382-5

**Published:** 2020-06-16

**Authors:** Anh Duc Nguyen, Gayoung Nam, Donghyuk Kim, Eun Yeol Lee

**Affiliations:** 1 Department of Chemical Engineering, Kyung Hee University, Yongin-si, Gyeonggi-do, 17104 South Korea; 2 School of Energy and Chemical Engineering, Ulsan National Institute of Science and Technology (UNIST), Ulsan, 44919 South Korea

**Keywords:** Adaptive laboratory evolution, C1 assimilation, Methanotroph, Pyrophosphate-linked phosphofructokinase, Pyrophosphate metabolism

## Abstract

**Background:**

Methanotrophs is a promising biocatalyst in biotechnological applications with their ability to utilize single carbon (C1) feedstock to produce high-value compounds. Understanding the behavior of biological networks of methanotrophic bacteria in different parameters is vital to systems biology and metabolic engineering. Interestingly, methanotrophic bacteria possess the pyrophosphate-dependent 6-phosphofructokinase (PPi-PFK) instead of the ATP-dependent 6-phosphofructokinase, indicating their potentials to serve as promising model for investigation the role of inorganic pyrophosphate (PPi) and PPi-dependent glycolysis in bacteria. Gene knockout experiments along with global-omics approaches can be used for studying gene functions as well as unraveling regulatory networks that rely on the gene product.

**Results:**

In this study, we performed gene knockout and RNA-seq experiments in *Methylotuvimicrobium alcaliphilum* 20Z to investigate the functional roles of PPi-PFK in C1 metabolism when cells were grown on methane and methanol, highlighting its metabolic importance in C1 assimilation in *M. alcaliphilum* 20Z. We further conducted adaptive laboratory evolution (ALE) to investigate regulatory architecture in *pfk* knockout strain. Whole-genome resequencing and RNA-seq approaches were performed to characterize the genetic and metabolic responses of adaptation to *pfk* knockout. A number of mutations, as well as gene expression profiles, were identified in *pfk* ALE strain to overcome insufficient C1 assimilation pathway which limits the growth in the unevolved strain.

**Conclusions:**

This study first revealed the regulatory roles of PPi-PFK on C1 metabolism and then provided novel insights into mechanism of adaptation to the loss of this major metabolic enzyme as well as an improved basis for future strain design in type I methanotrophs.

## Background

Methanotrophs, prokaryotes that use C1 compounds, such as methane or methanol, as the sole carbon and energy source, have drawn increasing attraction as promising industrial biocatalysts for bioconversion of methane and methanol to commercially relevant products [1–4]. In order to develop more efficient biocatalyst for C1 bioconversion, broadening knowledge on C1 metabolism is crucial. Multiple advantageous metabolic capabilities including high growth rate, well-established genetic toolbox, and availability of multi-omics studies made type I methanotrophs more attractive recently [1]. Type I methanotrophs mainly avail RuMP cycle for C1 assimilation in which formaldehyde condensed with ribulose monophosphate to produce fructose-6-phosphate, which is in turn incorporated into glycolytic pathways, such as Embden–Meyerhof–Parnas (EMP) and Entner–Doudoroff (ED) pathways [4, 5]. Some gammaproteobacterial methanotrophs use the EMP pathway as the main assimilation pathway for C1 substrates, which improves achievable carbon and energy yields compared to the ED pathway [6]. However, our fundamental knowledge of C1 assimilation of methanotrophs is still limited, particularly for pMMO regulation, energy mechanism, carbon flux regulation, and more. In general, metabolic pathways downstream of the primary methane assimilation are poorly understood, and such that knowledge on how methanotrophic bacteria adapt to different cultivation conditions are very restricted [1]. These gaps in knowledge should be investigated and be addressed in order to improve metabolic engineering strategies [1, 4]. *Methylotuvimicrobium alcaliphilum* 20Z, formerly known as *Methylomicrobium alcaliphilum* 20Z, is a model obligate methanotroph and it has been studied and developed to serve as an advantageous industrial biocatalyst for methane bioconversion [7–9]. Systems biology oriented knowledge-bases in accompany with the repositories of conventional genetic toolboxes in this model provide opportunities for a better understanding of central metabolism on different nutritional conditions for methanotrophs and further to guiding metabolic engineering strategy to produce relevant biochemical products [10].

It has been reported that intracellular inorganic pyrophosphate (PPi) concentrations in methanotrophs are 20 times higher than in *Escherichia coli* and low ATP concentration was observed in the methanotrophs [11]. Moreover, extremely low inorganic pyrophosphatase activity has been detected [12], suggesting an important role of PPi and PPi-dependent enzymes in the methanotrophic metabolism. Interestingly, *M. alcaliphilum* 20Z has pyrophosphate-linked phosphofructokinase (PPi-PFK) for glycolysis instead of the classical glycolytic enzyme ATP-dependent 6-phosphofructokinase (ATP-PFK). Among several PPi-dependent enzymes in methanotrophs, this enzyme could be a model to elucidate possible roles of PPi and PPi-dependent glycolysis in microorganisms [6, 12]. PPi-PFK is the starting point of the EMP pathway, and it catalyzes interconversion between fructose 6-phosphate (F6P) and fructose 1,6-bisphosphate (FBP), which might involve in PPi recycling to economize ATP synthesis as well as control the PPi level. Thus, in-depth investigation of PPi-PFK as well as other PPi-dependent enzymes can uncover more complex metabolic roles of PPi-pools, its biosynthesis, and cell energetics in methanotrophs. The utility of gene knockout approach has been proven useful to investigate the structure and dynamics of metabolic networks, particularly by observing responses to knockouts of metabolic enzymes and global regulators. Such that, the primary objective of this study is a comprehensive characterization of PPi-PFK in *M. alcaliphilum* 20Z with genetic modification, transcriptomic analysis, and network analysis with a genome-scale model.

Since regulation of cellular metabolism is pivotal in responding and adapting to changing environments, understanding the mechanisms of microbial adaptive evolution under such conditions in comparison to the wild-type and specific knock-out strains can shed light on functions of key metabolic genes and their effect propagating through the whole metabolic network. The deletion of such key regulator or metabolic enzyme could lead to redistribute of metabolic fluxes in the cell, which can settle down in using alternative pathways and regulation [13–15]. Adaptive laboratory evolution (ALE) is a powerful approach in which microbe is cultured continuously for many generations, subsequently improving its fitness through natural selection. The end-point mutants are then re-sequenced and characterized to identify genetic mutations and to investigate mechanistic insights with multiple-omics analysis [13–15]. ALE approach has been widely used not only to study the adaptation of the cells to shifting environmental conditions or toxic chemicals but also to investigate the adaptive responses to genetic perturbations such as loss of major metabolic enzymes [13–15]. This approach could provide valuable knowledge for both evolutionary and metabolic research. However, there reports on ALE in methanotrophs have been scarce. Thus, the study of PPi-PFK in the central metabolism of methanotroph with genetic perturbation for *pfk* and following ALE experiments was performed in hope to understand the metabolic roles of *pfk* in methanotrophs. In addition, transcriptomic analysis and network analysis were performed to gain insights on the fundamental roles of PPi-PFK in C1 assimilation for methanotrophs.

## Results

### PPi-PFK is required for efficient methanotrophic growth of *M. alcaliphilum* 20Z

**Fig. 1:**
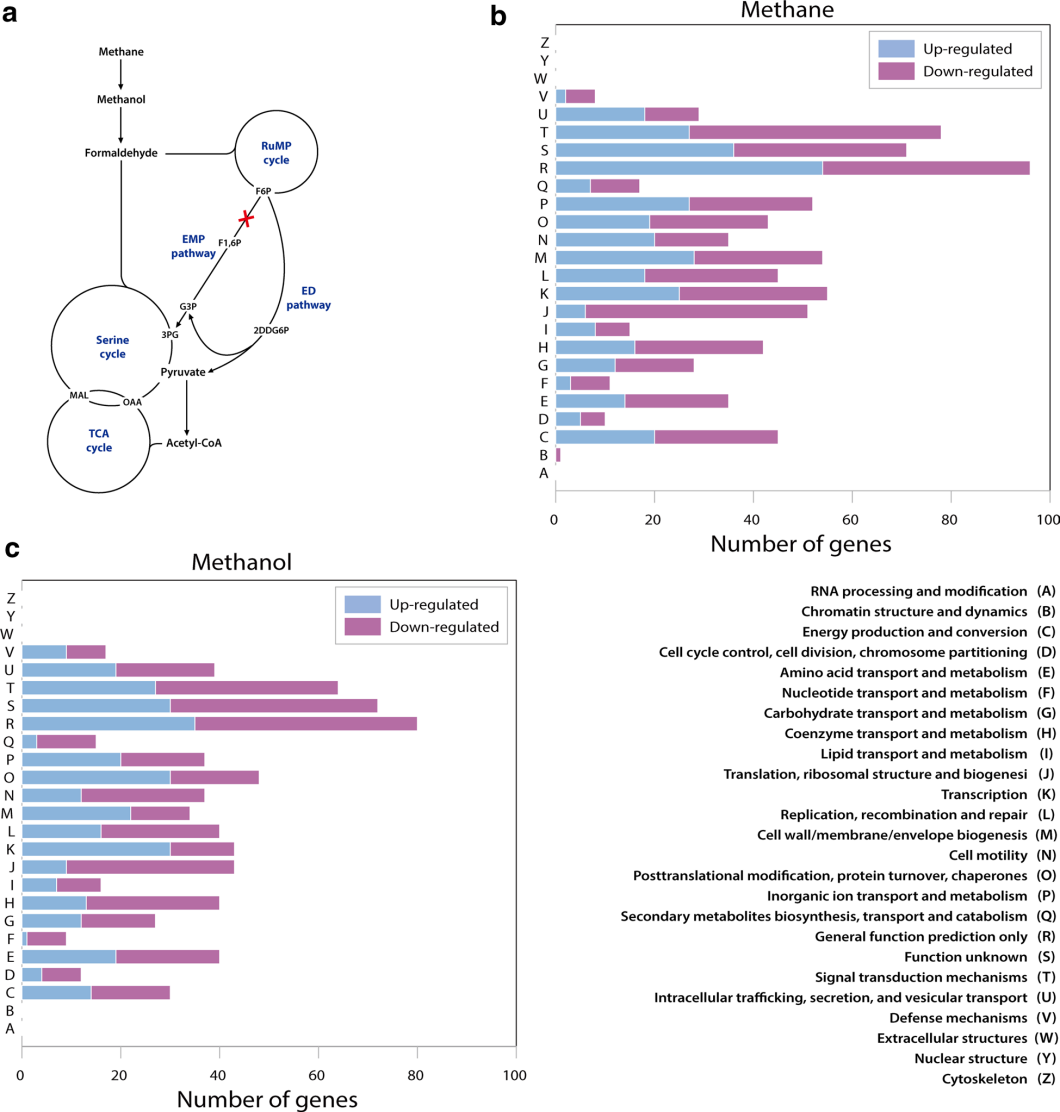
Central metabolism of *M. alcaliphilum* 20Z with pyrophosphate-linked phosphofructokinase (PPi-PFK) were deleted by the gene knockout, PPi-PFK phosphorylates fructose 6-phosphate to fructose 1,6-biphosphate in glycolysis pathway (**a**), Clusters of Orthologous Groups (COGs) analysis of differentially expressed genes when *pfk* was knockout on methane (**b**) and methanol (**c**)

It was postulated that methane assimilation in *M. alcaliphilum* 20Z is coupled with the pyrophosphate-mediated glycolytic pathway where the major fraction of cellular pyruvate comes from the Embden–Meyerhof–Parnas (EMP) pathway [6] (Fig. 1a). In order to assess the metabolic role and its impact of PPi-PFK on methane and energy metabolism of *M. alcaliphilum* 20Z under different culture conditions, a *pfk* mutant strain was constructed using marker-exchange protocol [7, 10]. After isolation of the *pfk* mutant on the selective plate, it was confirmed with Sanger sequencing and PCR [7, 10]. To evaluate the physiological effect of PPi-PFK, the *pfk* mutant was then cultured in liquid culture using NMS medium and the growth rate of Δ*pfk* was monitored on 2 different carbon sources, methane and methanol. During cells growth on both carbon sources, the Δ*pfk* showed reduced growth with longer doubling time and lag phase compared to wild-type (Additional file 1: Figure S1A). This observation confirmed that PPi-PFK is apparently involved in assimilating the carbon source and shed light on its possible role in methane and methanol metabolism of *M. alcaliphilum* 20Z. Interestingly, *pfk* knock-out decreased growth rate more severely on methanol (Additional file 1: Figure S1B), suggesting *pfk* might have different regulatory implication in utilizing different carbon sources.

In silico growth rate as well as metabolic flux distribution of wild-type and PPi-PFK knockout on methane and methanol was calculated with the genome-scale metabolic model (GSM) *i*IA407 and MCMC (Markov Chain Monte Carlo) sampling (Additional file 2: Table S1). The flux analysis with the GSM of *M. alcaliphilum* 20Z indicated that the metabolic reaction catalyzed by PPi-PFK can carry very high flux when cells were grown on both methane and methanol. Moreover in silico simulation with PPi-PFK deletion predicted a substantial decrease in the growth rate, agreeing with our experimental result (Additional file 1: Figure S1C, D).

### Transcriptome change in central metabolism of *pfk* knockout *M. alcaliphilum* 20Z on methane

**Fig. 2:**
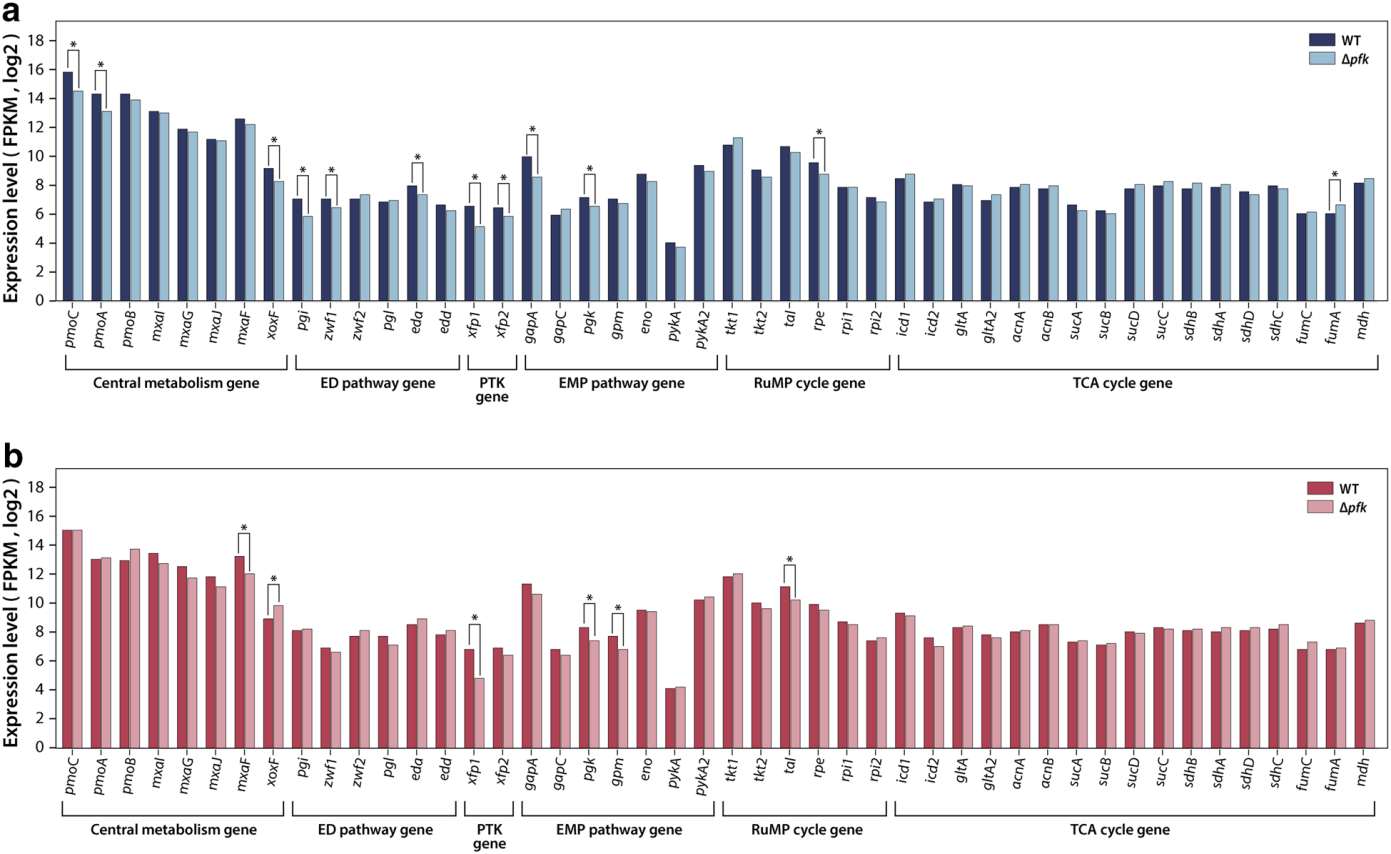
Comparison expression level of genes in the central metabolism of wild-type and Δ*pfk* growth on methane (**a**) and methanol (**b**). *Significantly different expression of genes between wild-type and Δ*pf*k (*P* < 0.05)

In order to further investigate the metabolic role of *pfk* in the central carbon metabolism, transcriptome analysis with RNA-seq was performed for *M. alcaliphilum* 20Z wild-type and Δ*pfk* knock-out strains on methane. During growth on methane, the expression level of 898 genes was changed significantly when *pfk* was removed: 475 genes down-regulated and 423 genes up-regulated (Additional file 3: Table S2). Functional analysis with Clusters of Orthologous Groups (COG) highlighted that differentially expressed genes have functions statistically enriched in signal transduction mechanism, inorganic ion transport/metabolism and cell wall/membrane/envelope biogenesis, suggesting deletion of *pfk* has effects on a broad range of gene functions (Fig. 1b). Then closer attention was paid to the transcriptional response of metabolic genes in the central metabolic pathways of *M. alcaliphilum* 20Z grown on methane. As a severe growth decrease of the *pfk* null mutant has implied on methane, a significant number of metabolic genes of central metabolic pathways showed differential expression (Additional file 3: Table S2). In agreement with this observation from RNA-seq analysis, RT-qPCR also showed the downregulation of several key genes in C1 assimilation pathway (Additional file 1: Figure S2A and S2B). In particular, the expression level of *pmo*, which encoded methane monooxygenase, was extremely down-regulated which was 1.4- to 2.5-fold changes, indicating PPi-PFK of *M. alcaliphilum* 20Z might be related directly or indirectly to the regulation of *pmo* (Fig. 2a) with unknown mechanisms. Another downstream gene for methane oxidation *xoxF*, lanthanide-dependent methanol dehydrogenase, contributes to the methanol oxidation process and it was down-regulated 1.8-fold changes as well. In contrast, the expression level of calcium-dependent methanol dehydrogenase, *mxaFJGI*, did not change significantly (Fig. 2a). Furthermore, formaldehyde-activating enzyme (*fae*) and formate dehydrogenase (*fdh*), which are the main enzymes for formaldehyde oxidation, were significantly down-regulated 2.7- and 7-fold, respectively (Additional file 3: Table S2).

The expression of genes in the C1 assimilation pathway changed significantly in *M. alcaliphilum* 20Z grown on methane when *pfk* was deleted. Metabolic enzymes in the ED pathway, such as glucose-6-phosphate isomerase (*pgi*), glucose-6-phosphate dehydrogenase (*zwf*) and keto-hydroxyglutarate aldolase (*eda*), were down-regulated 2.3-, 1.5-, and 1.5-fold, respectively, when *pfk* was knocked out. Interestingly, the significant downregulation was also observed for the genes in the EMP pathway and the serine cycle in Δ*pfk*. In detail, the expression of glyceraldehyde-3-phosphate dehydrogenase (*gapA*) and phosphoglycerate kinase (*pgk*) in EMP pathway were decreased 2.9-, and 1.5-fold, respectively; the expression of ribulose-phosphate 3-epimerase (*rpe*) belonging to RuMP cycle were downregulated 1.7-fold, and the expression of phosphoketolase (*xfp*) in phosphoketolase pathway were downregulated 2.8-fold. Additionally, the expression of glycerate 2-kinase (*gck2*), methylenetetrahydrofolate dehydrogenase (*mtdA*), serine-glyoxylate aminotransferase (*sga*) which belong to serine cycle were downregulated 1.8-, 2-, and 1.8-fold, respectively (Fig. 2a). However, most of the genes in the TCA cycle did not change, except for slightly up-regulated *fumA*. These responses in the central metabolic pathway along with growth defect on Δ*pfk* indicated that metabolic fluxes through the core carbon metabolic pathways were changed when *pfk* was deleted, suggesting its metabolic importance in methane assimilation in *M. alcaliphilum* 20Z.

### Different transcriptional response of PPi-PFK knockout on methanol

Compared to methane, the growth rate of *M. alcaliphilum* 20Z on methanol is much higher [16]. Moreover, it was reported that *Methylotuvimicrobium* species could accumulate a large amount of glycogen up to 30% dry cell weight and formate when cells are grown on methanol [10, 17]. Interestingly, our current study revealed that C1 assimilation pathways and energy regulatory mechanisms were regulated in a different manner for different carbon sources including methane and methanol in *M. alcaliphilum* 20Z [10]. As noted above, *pfk* deletion decreased growth rate more severely on methanol, suggesting it might possess different regulatory roles in C1 metabolism on methanol compared to methane (Additional file 1: Figure S1B). In order to evaluate transcriptome change on different carbon sources and *pfk* deletion, RNA-seq was performed with *M. alcaliphilum* 20Z wild-type and Δ*pfk* on methanol. As a result, 880 genes were identified with significant changes in transcription level of Δ*pfk* compared to wild-type grown on methanol: 481 genes down-regulated and 399 genes up-regulated (Additional file 4: Table S3). Functional analysis with COG was performed and revealed that differentially expressed genes have enriched functions in signal transduction mechanisms, post-translational modification, and protein turnover/chaperone functions (Fig. 1c).

As expected, comparative transcriptomic analysis between Δ*pfk* and wild-type on methanol showed differences in expression patterns of genes in central metabolism compared to methane. As for the C1 oxidation pathway, the expression of *pmo* did not change between Δ*pfk* and wild-type on methanol (Fig. 2b). When substrate shifts from methane to methanol, *pmo* was extremely down-regulated in wild-type because methane is one of key enhancer factors for *pmo* expression [18]. Not similar to that of wild-type, this effect did not occur in Δ*pfk* whereas similar expression of *pmo* was observed on methane and methanol (Additional file 1: Figure S3A, B). This observation supported our previous hypothesis that *pfk* might contribute to the regulation of the *pmo* operon. In addition, genes related to methanol oxidation also showed different expression patterns on methanol compared to methane when *pfk* was knocked out. While the expression of *mxaF* was not changed significantly on methane, the expression of *mxaF* was significantly down-regulated which was approximately 2.3-fold changes on methanol (Fig. 2b). Similar to that of *mxaF*, expression of genes related to pyrroloquinoline biosynthesis *pqqB* and *pqqC*, which provide cofactors for methanol oxidation, also down-regulated (Additional file 4: Table S3). In contrast, *xoxF* was slightly up-regulated (1.9-fold change) on methanol (Fig. 2b). In the formaldehyde oxidation pathway, expression of *fdh*, which encodes an enzyme to convert formate to CO_2_ with the generation of NADH, was greatly decreased which was sevenfold changes.

The expression of the genes for the C1 assimilation pathway was also affected on *pfk* knockout when cells were grown on methanol. Not similar to growth on methane where gene expression for the ED pathway was repressed, expression of these ED pathway encoding genes did not change significantly on methanol. In addition, the down-regulation of some key genes in EMP pathway, RuMP cycle, and phosphoketolase pathway were observed on methanol. The expression of *pgk* and phosphoglycerate mutase (*gpm*) in EMP pathway were greatly decreased 1.8- and 1.8-fold, respectively. The expression of transaldolase (*tal*) belonging to RuMP cycle and *xfp* from phosphoketolase pathway were also downregulated which were 1.9- and 4-fold, respectively (Additional file 4: Table S3). Furthermore, expression of *sga*, *mtdA*, *hpr2* (D-glycerate dehydrogenase) and *mclA1* (CoA ester lyase) which belong to the serine cycle also down-regulated which were 2.4-, 1.7-, 1.6- and 2-fold, respectively. Most of the genes in the TCA cycle did not change significantly except for slight down-regulation of succinate-semialdehyde dehydrogenase (*gabD*) (Fig. 2b).

In summary, knock-out of *pfk* resulted in down-regulation of many genes in the central metabolic pathways on methanol, whereas the overall transcriptional landscape on methanol were different compared to growth on methane, highlighting the differences of metabolic roles of PPi-PFK for methane and methanol metabolism in *M. alcaliphilum* 20Z.

### Recovery of growth fitness of Δ*pfk* with ALE and identification of key mutations

**Fig. 3:**
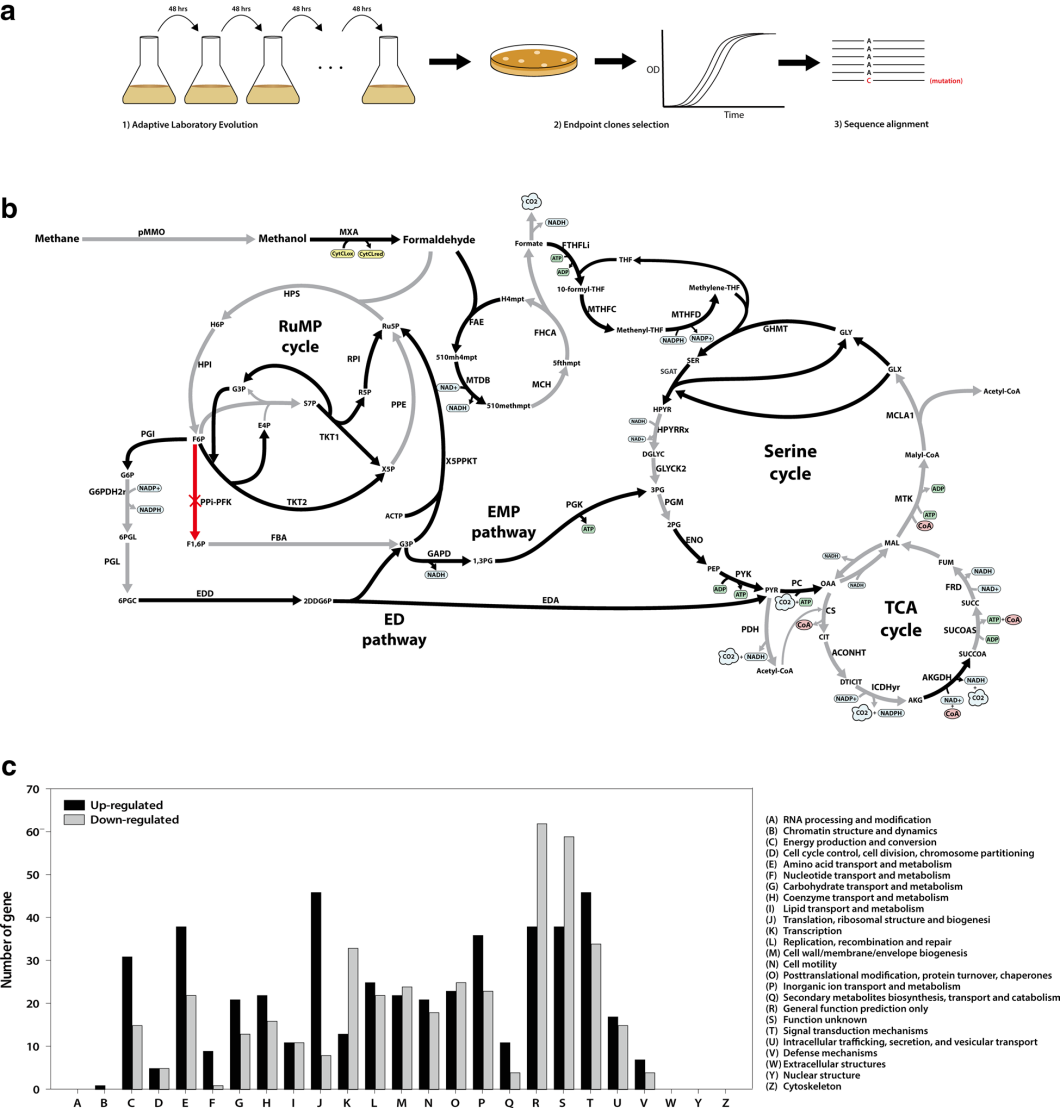
Adaptive evolution approach used to isolate evolved strains and characterization of evolved strains by NGS-based studies (**a**). The central metabolism of Δ*pfk* ALE strain grown on methane. Genes highlighted in black were significantly up-regulated in Δ*pfk* ALE strain compared to unevolved strain (**b**), Clusters of Orthologous Groups (COGs) categories of up-regulated and down-regulated genes in *pfk* ALE strain compared to unevolved strain (**c**)

Deletion of *pfk* in *M. alcaliphilum* 20Z results in much slower growth than wild-type. Adaptive laboratory evolution (ALE) experiments with Δ*pfk* resulted in evolved strains with significantly recovered growth rate (Fig. 3a, Additional file 1: Figure S4A). The evolved strains had similar growth rates as to the wild-type on both methane and methanol (Additional file 1: Figure S4B). To assess the genetic basis of the growth rate recovery, whole-genome resequencing was performed. Resequencing sequence reads were mapped onto the *M. alcaliphilum* 20Z reference genome to identify mutations in two ALE strains. Not similar to *E. coli* which can adapt to the loss of a major metabolic gene product such as PGI with only a handful of mutations [19], *M. alcaliphilum* 20Z overcame the loss of key regulator with a high number of mutations where 320 nonsynonymous substitution mutations were observed (Additional file 5: Table S4), including 219 mutations (68.4%) spanning in 40 intergenic regions and 101 mutations (31.6%) spanning in 56 ORFs. Interestingly, among these substitution mutations, 295 mutations (92.5%) were presented in both evolved strains. The similarity in mutation profiles between these two ALE strains suggested the unique adaptive responses to the loss of major metabolic enzyme, PPi-PFK, in *M. alcaliphilum* 20Z. Functional analysis with COG was also performed with the genes with mutations, and it was found that those genes have functions enriched in diverse categories including signal transduction mechanisms and lipid transport and metabolism (Additional file 1: Figure S5). Interestingly, among mutated points, we found a large region of 22 kb (from MEALZ_RS01220 to MEALZ_RS01310) spanning 17 mutated ORFs. In this region, there are three genes encoding sulfate transport via the ABC system (*cysAW*, *sbp*) and four genes related to fatty acid biosynthesis (*fab*) (Additional file 5: Table S4). Moreover, two endpoint strains have a single-nucleotide mutation in an RNA polymerase subunit beta (*rpoC*) (C→A; D170Y) (Table 1), which might implicate in the control of transcription pausing and elongation [20].

**Table 1:** Key genetic changes in evolved strains

Locus_Tag	Gene	Product	Reference sequence location	Mutation
MEALZ_RS01065	*hppA*	Sodium-translocating pyrophosphatase	223,987	N30S (AAC→AGC)
MEALZ_RS01220	*cysB*	LysR family transcriptional regulator	264,570	E154D (GAA→GAC)
MEALZ_RS01235	*cysA*	Sulfate ABC transporter ATP-binding protein	268,089 268,091 268,121	K19D (AAA→GAC) K19D (AAA→GAC) S9G (AGC→GGC)
MEALZ_RS01240	*cysW*	Sulfate ABC transporter permease subunit CysW	269,090 269,198 269,207	S63A (TCT→GCT) L27M (CTG→ATG) R24C (CGC→TGC)
MEALZ_RS01250	*sbp*	Sulfate ABC transporter substrate-binding protein	270,878	K267N (AAA→AAT)
			271,623	A19V (GCT→GTT)
MEALZ_RS01295	*fabG*	3-Oxoacyl-ACP reductase FabG	282,479 282,486	Y78H (TAC→CAC) A80V (GCT→GTT)
MEALZ_RS06915	*folD*	Bifunctional methylenetetrahydrofolate dehydrogenase/methenyltetrahydrofolate cyclohydrolase FolD	1,598,870	D87E (GAT→GAA)
MEALZ_RS16400	*rpoC*	DNA-directed RNA polymerase subunit beta	3,871,938	D170Y (GAT→TAT)
MEALZ_RS17985		Outer membrane lipoprotein carrier protein LolA	4,233,780	A183V (GCC→GTC)
MEALZ_RS18010		Transcriptional regulator	4,241,883	S389G (AGT→GGT)

In the carbon metabolism, single nucleotide mutation in the bifunctional methylenetetrahydrofolate dehydrogenase/methenyltetrahydrofolate cyclohydrolase encoded by *folD* (A→T; D87E) was discovered, and it may contribute to NADPH production and C1 assimilation via the folate pathway (Table 1). It was predicted that this pathway may carry more flux in the Δ*pfk* ALE strain, suggesting the mutation in *fodD* might enable a specific function. Although *M. alcaliphilum* 20Z possesses both of methenyltetrahydrofolate cyclohydrolase (*fchA*) and methylenetetrahydrofolate dehydrogenase (*mtdA*) for methylene tetrahydrofolate pathway, it also contains *folD* which encoded a bifunctional methylenetetrahydrofolate dehydrogenase/cyclohydrolase. The function of these genes have not been characterized in type I methanotrophs. All of these three genes were expressed, with slightly higher expression of *mtdA* and *fchA* than *folD* (Additional file 6: Table S5). The dehydrogenase activity of *folD* catalyzes NADP+-dependent oxidation of 5,10-methylenetetrahydrofolate to 5,10-methenyl-tetrahydrofolate and subsequently to 10-formyltetrahydrofolate (10-CHO-THF) by the cyclohydrolase activity [21]. In order to confirm if the mutation point of *folD* might be led to change in its binding activity, multiple alignment was performed among *folD* from wild-type *M. alcaliphilum* 20Z, ALE strain and other well-studied *folD* with available crystal structures [21]. Even the 3D structures of FolD from different species are similar, they possess different specific activities and affinities for the inhibitors. The amino acid at position 87 is diverse among these species (D87, E87, and A87) so that substitution from D87 to E87 in *folD* could change the binding activity of this particular enzyme (Additional file 1: Figure S6). Given the importance of this enzyme for co-factors regulation, the reported mutation from this study may provide valuable new candidates for rationally manipulating *folD* activity in metabolic engineering of methanotrophs. Additionally, seven mutations were also detected in the intergenic region upstream of *fodD* which might be related to the transcriptional regulation of this gene. Similar to *fodD*, sodium‑translocating pyrophosphatase (*hpp*), which is involved in energy regulation mechanism, was also found to gain one mutation in the ORF (A→G;N30S) (Table 1). Notably, two more mutations were found in the upstream intergenic region of *hpp* where 1-bp deletion was identified. Transcriptome analysis between wild-type and one evolved strain was performed to further investigate how these mutations could enable the increasing growth rates from the initial state.

### Transcriptome analysis for mutated genes of ALE strain

Integration of mutation analysis with the evolved strains and functional analysis for the genes with mutations narrowed down possible genes that could render different phenotypes after adaptive evolution. First of all, a mutation of RNA polymerase subunit, *rpoC*, could be a potent rescue solution for the *pfk* deletion. In the previous report with *E. coli* adaptive evolution after deletion of a major enzymatic gene of the EMP pathway, *pgi*, the catalytic subunit of RNA polymerase, *rpoB*, was mutated to change the genome-wide transcriptional landscape [22]. Similar to RpoB, a mutation of RpoC might lead to transcriptional change of multiple enzymatic genes. Besides, mutations in two transcriptional regulators (locus tag: MEALZ_RS18010 and MEALZ_RS01220) which encoding for an uncharacterized transcriptional regulator and HTH-type transcriptional regulator CysB were also identified in each of the fast-growing evolved strains. It can be hypothesized that these mutations might contribute to growth recovery by modulating transcription level of alternative pathways in the ALE strains (Fig. 3b).

Mutations in transcriptional regulatory machineries including RNA polymerase subunits and transcription factors necessitated investigation of genome-wide transcriptome to compare the wild-type and evolved mutant strain, hoping to characterize the rewiring of central metabolism to overcome the deletion of *pfk*. As a result, transcriptome analysis with RNA-seq approach was carried out when cells were grown on methane and revealed expression change of 1040 genes, where 514 and 526 genes were up- and down-regulated in the ALE strain compared to the parental strain (Additional file 6: Table S5). Functional analysis with COG was then performed to show differentially expressed genes had enriched functions in signal transduction mechanisms followed by inorganic ion transport and metabolism (Fig. 3c).

Comparative transcriptomic analysis showed the global expression changes in central metabolism which might be associated with growth recovery in the ALE strain (Fig. 3b). The expression of *pmo* did not change between the unevolved and ALE strain which still lower than the expression level of *pmo* in the wild-type. Interestingly, the up-regulation of methanol oxidation enzymes including lanthanide-dependent methanol dehydrogenase (*xox*) and calcium-dependent methanol dehydrogenase (*mxa*) was observed in the evolved strain which were 1.6- and 1.7-fold, respectively (Additional file 6: Table S5). Likewise, RT-qPCR also showed the upregulation of these metabolic genes in the evolved strain (Additional file 1: Figure S4C). In agreement with this observation, evolved strain had slightly higher methanol uptake rate compared to the wild-type (Additional file 1: Figure S4D). Furthermore, the formaldehyde oxidation pathway also was activated in the ALE strain, where genes in the H4MPT, H4F pathways were up-regulated (Fig. 3b).

Activation of the formaldehyde oxidation pathway in the evolved Δ*pfk* strain brought more attention to investigate possible other changes in C1 assimilation pathway (Fig. 3a). First of all, activation of the ED pathway was observed with high up-regulation of key enzymatic genes such as *pgi*, *edd*, and *eda* (Fig. 3b, Additional file 1: Figure S4C). Without PPi-PFK in the core carbon pathways, the ED pathway might need to be activated accommodate carbon flux increase to produce sufficient G3P pool by making G3P from F6P (Fig. 3b). G3P, then, can subsequently enter the EMP pathway or the RuMP cycle. In agreement with this observation, in silico flux analysis with the GSM *i*IA407 and MCMC sampling also indicated an increasing flux thought the ED pathway when PPi-PFK was removed from the model (Additional file 2: Table S1). At the G3P node, the RuMP cycle via the non-oxidative PP pathway-which mainly provided C5 unit for formaldehyde assimilation, was up-regulated with significant increase in the transcription level of transketolase (*tkt*) and ribose 5-phosphate isomerase (*rpi*) which were 1.9- and 1.7-fold, respectively (Additional file 6: Table S5). In addition, the partial EMP pathway which further converts G3P to pyruvate was strongly activated with high up-regulation of glyceraldehyde-3-phosphate dehydrogenase (*gapA*) (3.6-fold), phosphoglycerate kinase (*pgk*) (2.6-fold), phosphopyruvate hydratase (*eno*) (1.8-fold) and pyruvate kinase (*pyk*) (2.1-fold) (Additional file 1: Figure S4C, Additional file 6: Table S5). Furthermore, the serine cycle, which interconnected with TCA and glycolysis pathway for acetyl-CoA formation, was also up-regulated with an increasing expression level of serine-glyoxylate aminotransferase (*sga*), serine hydroxymethyltransferase (*glyA*), bifunctional methylenetetrahydrofolate dehydrogenase/cyclohydrolase (*folD*) and formate-tetrahydrofolate ligase (*ftfL*) which were 3.2-, 1.6-, 1.9- and 1.5-fold (Additional file 6: Table S5). Consistent with the up-regulation of *folD*, some mutation points in the upstream region of *folD* was detected which might direct or indirect affect to transcription of this gene. It currently has been reported that the incomplete serine cycle only carries minor flux to acetyl-CoA production in *M. alcaliphilum* 20Z with a small amount of formate secretion [8]. However, higher secretion rate of formate from the ALE strain was observed when compared to wild-type (Additional file 1: Figure S4D), which explains the formaldehyde oxidation pathway and the serine cycle might carry more flux to contribute the formation of reducing equivalents as well as acetyl-coA production when *pfk* is deleted [10]. In addition, the expression level of genes in the TCA cycle did not change significantly except slight up-regulation of *sucA*, and *sucB*. In summary, transcriptome analysis with the evolved Δ*pfk* strain illustrated possible activation of bypass pathway through the ED–EMP pathway. This result agreed with in silico flux analysis with the GSM and the mutation analysis with the ALE strain.

### Differential regulation of PPi metabolism in the ALE strain

**Fig. 4:**
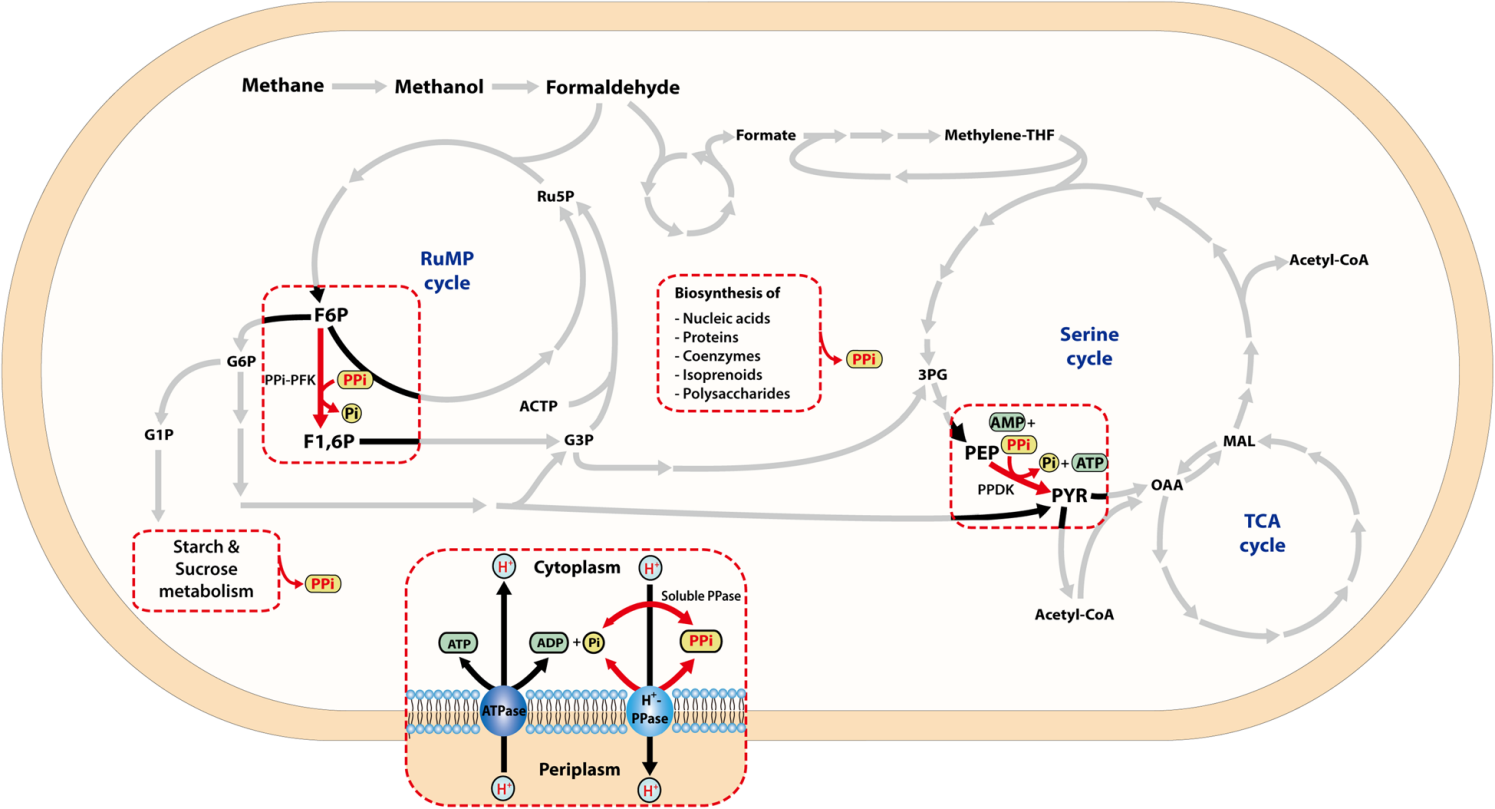
PPi-dependent C1 metabolism in *M. alcaliphilum 20Z*. *PPi-PFK* PPi-dependent 6-phosphofructokinase, *PPDK* pyruvate, phosphate dikinase

Inorganic pyrophosphate (PPi) is a by-product of several anabolic reactions such as biosynthesis of nucleic acids, proteins, coenzymes, isoprenoids, and oligo- and polysaccharides. Moreover, in *M. alcaliphilum* 20Z, the high accumulation of glycogen and sucrose suggested that the biosynthesis pathways of sucrose and glycogen might function as major sources to contribute to PPi generation (Fig. 4). It has been reported that intracellular PPi concentration in methanotrophs was 20 times higher than in *E. coli* while only low ATP concentration found which correlated with extremely low inorganic pyrophosphatase activity [12], suggested that energy-rich PPi molecules also play an important role in the energy metabolism in methanotrophs. The discovery of the activity of PPi-PFK along with a high intracellular concentration of PPi indicated that PPi-PFK could be one unique metabolic features of the energy generation mechanisms in methanotroph [11]. With PPi-dependent glycolysis pathway, the energy of PPi can be re-utilized to generate ATP, which might significantly increase the efficiency of one-carbon assimilation.

In the absence of PPi-PFK, PPi-dependent glycolysis pathway was repressed and could not re-utilized PPi efficiently, which might affect cell energetics, subsequently leading to decrease growth rate. So it was hypothesized that the PPi metabolism might change to adapt the absence of PPi-PFK in the ALE strain. To investigate the roles of PPi metabolism, we further confirmed the changes of others key PPi-linked enzymes in the ALE strain. Pyruvate phosphate dikinase (PPDK) catalyzes the reversible conversion of phosphoenolpyruvate (PEP) into pyruvate accompanied by ATP synthesis from AMP. With this enzyme, the PPi-dependent glycolysis pathway was completely reversible and energetically 2.5 times more efficient in comparison to the ATP-dependent glycolytic pathway [23]. Interestingly, the expression level of *ppdk* was increased by twofold in the ALE strain, suggesting that PPDK might take over the role of PPi-PFK to generate ATP via re-utilization of PPi (Fig. 3a, Additional file 6: Table S5). In addition, *M. alcaliphilum* 20Z possesses a membrane-bound proton-translocating H+-Ppase which might contribute to the regeneration of PPi from ATP. The predicted ratio of ATP hydrolysis: PPi formation for this enzyme is 1:3 and it was expected that these enzymes contribute to 25% of PPi pool upon active growth on methane in this strain [24]. Mutation analysis with resequencing showed that mutation points were found in the coding sequence of *hpp* as well as its upstream intergenic region, suggesting transcription regulation of *hpp* might change. In agreement with this, the transcription level of *hpp* in the ALE strain was significantly down-regulated, so that the mutations points in the intergenic region might directly affect transcriptional regulation of *hpp*. It is highly likely that the decreasing expression of *hpp* might contribute to ATP generation from PPi in the evolved strain.

## Discussion

Loss of function strategy with gene knockout has been broadly used in biology to identify the gene functions and their roles in the biological networks. In particular, this approach has long been used in the study of microbial metabolism by observing responses to knockouts of metabolic enzymes and global regulators. The primary objective of this study was the elucidation the functional role of PPi-PFK, a key regulatory step of carbon metabolism in type I methanotrophs, in different culture conditions by using gene knockout and transcriptome analysis with RNA-seq. PPi-PFK has been found in all aerobic gammaproteobacterial methanotrophs and kinetic properties of this enzyme was characterized recently [24]. It also was tempting to speculate that PPi-PFK could be related to energy generation regulation in type I methanotrophs [24]. In this knockout studied, it was observed that growth rate of *pfk* mutant was extremely decreased on both methane and methanol possibly due to insufficient C1 metabolism. PPi-mediated EMP pathway is the main route for C1 assimilation which mainly contributed to pyruvate synthesis [6]. Due to knockout of *pfk*, the preferential route of carbon flow becomes the ED pathway, where the strain recruits the reducing powers as well as key intermediates G3P and pyruvate for the system. However, as noted above, genes in the ED pathway were significantly down-regulated on methane. Likewise, the genes encoding for the partial EMP pathway, which converts G3P to pyruvate and generates NADH, ATP as well as RuMP cycle have been down-regulated. Thus, the decreasing growth rate may be due to the fact that the *pfk* knockout apparently disrupts the energy and reducing power balance. Type I methanotrophs possess sugar-phosphate dependent metabolic pathways which are similar to those in *E. coli*. The deletion of PGI to block the glycolytic pathway also led to decrease specific growth rate due to the imbalance of reducing power with overproduction of NADPH [14, 19].

When methanol is metabolized as a sole carbon source, the effect of *pfk* knockout is slightly different from that of the case used methane as a sole carbon source. The most striking difference is the decrease in growth rate as compared to methane, suggesting the important role of *pfk* during growth on methanol. Additionally, transcriptional responses of central metabolism showed different effects when *pfk* was inactivated on methanol. The C1 assimilation pathway via the RuMP cycle also was slightly different than that of methane, where the genes in the ED pathway did not change in Δ*pfk* on methanol. This implies that the ED pathway seems less important for growth on methanol. On methane, the ED pathway contributes less to methane assimilation where 25% of intracellular pyruvate comes from this pathway and 75% comes from the EMP pathway. However, the ratio of carbon flux through EMP and ED pathways could alter under different carbon sources. In our recent study, the roles of EMP and ED pathway on methanol-grown cells were investigated to show the EMP pathway still dominates over the ED pathway for C1 assimilation and the ED pathway carries very low carbon flux [10]. In addition, the accumulation of a large amount of glycogen on methanol could lead to higher PPi generation on methanol (Fig. 4) and PPi-mediated EMP appears to re-utilize PPi to generate energy and reducing power for cell growth. It has been found that the expression of the serine cycle was more activated on methanol and deletion of PPi-PFK showed more defects on this pathway, suggesting the operation of a functional serine cycle. In agreement with this observation, when shifting the substrate from methane to methanol, our current studies showed the activation of serine cycle on methanol-grown for energy regulation [10, 16, 18], while the serine cycle only carries negligible flux for growth on methane [8]. In summary, those results demonstrated the impact of *pfk* knockouts on the metabolic phenotype is more expressive and also showed to have a different influence on the metabolism of grown on methane and methanol.

On methane, the expression level of *pmo* along with *xoxF* showed a similar expression pattern when *pfk* was removed. Our previous study suggested that *xoxF* could contribute to the methane oxidation process in type I methanotrophs [18] by formation of an alternative structure of MDH-pMMO association with the binding of XoxF monomer to pMMO [25]. So that, *pfk* likely serve as regulatory effects to control this structure. However, these effects might occur due to the imbalance of PPi, which potentially serves as an alternative phosphoryl donor instead of ATP. The pMMO located in the membrane of methanotrophs which also possesses an electron transport chain (ETC) and ATP synthesis through oxidative phosphorylation. These two processes, methane oxidation and ETC, might occur simultaneously, suggesting a complicated regulatory network including these processes. It was previously suggested that the source of PPi for PPi-PFK may be energy-dependent PPi synthesis on the cell membrane [11]. The disruption of PPi-PFK reaction might change the cell membrane integrity, subsequently affects to pMMO as well as aerobic ATP synthesis through ETC.

In the next step, we further investigated how the recovery of growth rate in Δ*pfk* is enabled by genetic mutations and rewiring of the metabolic network. Comparison of genome, transcriptome, and phenotype of wild-type and Δ*pfk* ALE strains could provide insight into how *M. alcaliphilum* 20Z may adapt to the loss of a key metabolic enzyme, PPi-PFK. The fitness recovery might be obtained by global transcriptional regulation such as RNA polymerase component *rpoC* which could contribute to a rebalancing of the proteome to promote growth [26]. This led to increased flux through oxidative pentose phosphate and ED pathways for generation of reducing powers and G3P which subsequently enters RuMP cycle for C5 unit regeneration as well as go to the EMP pathway for pyruvate synthesis. The increasing carbon flux through oxidative pentose phosphate pathway could increase NADPH availability which is a key factor for enhancing the biosynthesis of secondary metabolites from methane using methanotrophic bacteria as a biocatalyst. Furthermore, the upregulation of ED pathway is also an advantage for production of isoprenoids via methylerythritol phosphate (MEP) pathway since pyruvate and G3P were generated simultaneously via ED pathway, providing a more balanced precursor pool for the MEP pathway [27]. Moreover, carbon flux likely also enters H_4_MPT, H_4_F, and serine cycle to generate more reducing powers as well as contribute to acetyl-CoA production which is advantageous to produce various products derived from intermediates from the serine cycle, such as amino acids and CoA derivatives. The availability of C1 assimilation pathway may enable flexibility when adapting to changing conditions. The mutation of this global transcription pattern has been found in *E. coli* through adaptive evolution reprogram in different conditions. For example, the mutation in *rpoC* improved metabolic efficiency to gain growth advantage from adaptation to glycerol as well as growth in minimal media [22, 28]. Besides, the adaptation of *E. coli* Δ*pgi* also was accompanied with mutations in *rpoC*.

Notably, it was also found that the fitness recovery also relieve an alternative PPi mediated glycolysis. Inorganic pyrophosphate created during biosynthetic polymerization reactions in most organisms is hydrolyzed by inorganic pyrophosphatase in order to thermodynamically favor the anabolic processed [11]. However, with the negligible activity of inorganic pyrophosphatase found in methanotroph, the use of PPi instead of ATP as a phosphoryl donor in some key metabolic reactions such as PPi-PFK may provide a solution for economizing ATP synthesis, regulation of hexosephosphates and triosephosphates interconversion as well as PPi levels in cells [11, 12]. With regard to the previous studies, high flexibility at the key glycolytic steps could provide some advantages for methanotrophs, allowing metabolism to adapt to different energy and carbon sources. With the loss of PPi-PFK, energetics of cells was dramatically affected, and glycolytic steps turned into another strategy to adapt by using alternative PPi-dependent enzyme in glycolysis pathway PPDK for ATP synthesis and coupled with PPi recycling.

## Conclusion

In summary, this study highlighted the importance of PPi-PFK on C1 metabolism of *M. alcaliphilum* 20Z, which is considered as an industrially promising candidate for bioconversion of methane and methanol into value-added products. Additionally, we first demonstrated the combination of adaptive evolution experiment and -omic analysis to study the regulatory mechanism in the Δ*pfk* ALE strain. While whole-genome resequencing enabled the mutation discovery, and transcriptomic analysis coupled with genome-scale modeling revealed the metabolic pathways underlying the evolved phenotypes. This knowledge could provide novel strategies for evolution-based metabolic engineering for methanotrophs.

## Methods

### Bacterial growth conditions

The strains used in this study were grown in a nitrate mineral salt (NMS) medium [29]. Liquid pre-cultures of *M. alcaliphilum* 20Z were grown in 180 mL baffled-flask with 10 mL NMS with a supplement of 50% methane (v/v) as a sole carbon source at 30 °C and 230 rpm agitation. Fresh NMS medium of 50 mL was then inoculated with the pre-culture in a 500 mL baffled flask sealed with a screw cap with methane supplied to a final concentration of 50% (v/v) by gas substitution using a gas-tight syringe. For methanol, *M. alcaliphilum* 20Z was cultured in the same medium containing 1% methanol instead of methane for a carbon source. The initial optical density of each culture was set to OD_600_ = 0.01 for growth rate experiments. All culture experiments were performed with at least two biological duplicates. For strain construction, gentamicin was used at final concentrations of 10 μg/mL.

### Total RNA isolation and sequencing

For RNA-seq experiments, all cultures were grown in biological duplicates for subsequent RNA extraction and sequencing steps. For sequencing library preparation, 5 mL of microbial culture broth containing either methane- or methanol-grown culture, grown as described above, in mid-exponential phase were harvested (OD ~ 0.6–0.7 for methane growth and OD ~ 1–1.2 for methanol growth) and immediately mixed with 10 mL of bacterial reagent RNAprotect (Qiagen, Hilden, Germany) for ensuring reliable gene expression and gene-profiling data. Total RNA was then extracted using RNeasy Mini Kit along with On-Column DNase Digestion using RNAse-Free DNase Set (Qiagen, Hilden, Germany) following the manufacturers’ protocols. Total RNA quality and quantity were measured using an RNA 6000 Nano kit with the Agilent Bioanalyzer 2100 (Agilent Technologies, CA, USA) with an RNA Integrity Number (RIN) value greater than or equal to 7. In addition, total RNA samples were checked on 1.3% agarose gel to validate no DNA contamination (Additional file 1: Figure S7).

Next, for mRNA enrichment, rRNA was removed using the Ribo-Zero rRNA removal kit for gram-negative bacteria (Epicentre, Madison, WI, USA), and the remaining RNA was used for creating the sequencing library using the TruSeq Stranded Total RNA Sample Prep Kit (Illumina, USA) according to the manufacturer’s instructions. Transcriptome sequencing was conducted using the Illumina/Hiseq-2000 RNA sequencing platform with ~ 30 million/sample PE101 reads by Macrogen (Korea) **(**Additional file 7).

### Quantification of differentially expressed genes

The quality of the raw sequence data was examined with FastQC (http://www.bioinformatics.babraham.ac.uk/projects/fastqc/) for further quantitative analysis as previously described [30]. Sequencing reads were mapped onto the genome sequence of *M. alcaliphilum* 20Z (NC_016112 for the chromosome and NC_016108 for the plasmid). Bowtie toolbox [31] was used with a maximum insert size of 1000 bp and with two maximum mismatches after trimming 3 bp at the 3′ ends with other default option. The sequence alignment map (SAM) files were post-processed with SAMTools (http://samtools.sourceforge.net/) to be converted to the binary alignment/map (BAM) files, and then were sorted and indexed. Indexed and sorted BAM files were fed to Cuffdiff [32] to calculate differential expression (with default options including geometric normalization method and a library type of dUTP RNA-seq). Genes with differential expression of log2 fold change ≥ 1.0 and false discovery rates (FDR) value ≤ 0.01 were considered as differentially expressed genes in this study.

### DNA sequencing

Genomic DNA was isolated using a Promega Wizard DNA purification kit (Promega, Madison, WI, USA) following the manufacturers’ protocols. DNA was quantified by using a Qubit dsDNA high-sensitivity assay. Paired-end sequencing libraries were generated and sequenced with Illumina Hiseq-2000 sequencing platform (Macrogen, Korea). The breseq pipeline [33] was used to map sequencing reads and identify mutations relative to the genome sequence of *M. alcaliphilum* 20Z (NCBI accession NC_016112 for the chromosome and NC_016108 for the plasmid).

### Calculation of in silico metabolic flux with FBA analysis and MCMC sampling

To calculate in silico metabolic flux, FBA analysis, and MCMC sampling was performed with *M. alcaliphilum* genome-scale model *i*IA407 [8] and COBRApy [34] as previously described [35]. The *i*IA407 model is available on the following web-site: http://sci.sdsu.edu/kalyuzhlab/. The flux distribution for each reaction in the model was determined using Markov chain Monte Carlo (MCMC) sampling [36]. The uptake rates for all of the carbon sources, methane and methanol, were constrained to the model as 11.7 mmol/gDW/h. The biomass objective function (BOF) was set at a lower bound of 95% of the optimal growth rate as calculated by FBA. Thus, the sample flux distributions by MCMC sampling method could be represented sub-optimal flux distributions. MCMC sampling was performed by *optGpSampler* sampling algorithm with 100,000 of feasible fluxes. After normalization of average flux value against BOF, flux value each reaction was used for further analysis.

### Analytical analysis of culture supernatants

The culture supernatant was separated from cells by centrifugation. Formate and methanol concentrations were detected and measured using HPLC (Jasco Co., Easton, USA) with an RI detector and an Aminex HPX-87H organic acid column (Bio-Rad, Hercules, USA). Sulfuric acid (0.005 M) was used as the mobile phase at 60 °C with a flow rate of 0.7 mL/min. All solutions were filtered through a 0.2 µm membrane before use. The concentrations of formate and methanol were calculated by regression analysis compared to known standards.

### Adaptive laboratory evolution

Evolution of *M. alcaliphilum* 20Z Δ*pfk* was conducted in 50 mL of NMS medium with a supplement of 1% methanol in baffled flasks as the batch cultures. At the start of evolution, Δpfk was grown in 50 mL NMS medium in 500 mL baffled flasks at 30 °C and 230 rpm in 48 h and the cultures were then transferred to fresh medium for adaptive evolution at an initial OD_600_ of 0.01. Cells were grown reach to exponential phase, then diluted by serial transfer into fresh medium. Each transfer into the fresh medium was started at an OD_600_ of 0.01. This process, in batch growth and serial dilution, was conducted for 40 days for a total of 20 transfers to fresh medium. The strain from the last transfer was streaked out on NMS medium and incubated for 2 days. Then, 10 endpoint clones were selected to further confirm the growth rate.

### COG functional enrichment

DE genes were categorized according to their annotated clusters of orthologous groups (COG) category. Functional enrichment of COG categories DE genes was determined by performing a hypergeometric test, and P-value < 0.05 was considered significant.

### RT-qPCR

Total RNA samples were directly used for RT-qPCR experiments. The primers used for RT-qPCR provided in Additional file 8: Table S6. cDNA was synthesized from 500 ng total RNA using a RevertAid First Strand cDNA Synthesis Kit (Thermo Scientific, Waltham, USA) and used as a template for RT-qPCR. RT-qPCR was performed by using GoTaq qPCR Master Mix (Promega, Madison, USA) in CFX Connect Real-Time PCR Detection System (Biorad, Hercules, USA). The cycle threshold (Ct) value for each gene was determined and normalized to the *rpoD* housekeeping gene. The relative expression between two samples was calculated by using the ΔΔCt method [37].

## Supplementary information

**Additional file 1.** Additional figures and legends supporting the results described in text.

**Additional file 2.** Nomarlized flux distribution of wild-type and ∆*pfk* by MCMC sampling.

**Additional file 3.** Differentially expressed genes of *Methylotuvimicrobium alcaliphilum* 20Z wild-type and Δ*pfk* grown on methane.

**Additional file 4.** Differentially expressed genes of *Methylotuvimicrobium alcaliphilum* 20Z wild-type and Δpfk grown on methanol.

**Additional file 5.** Mapped genetics changes in ALE strains.

**Additional file 6.** Differentially expressed genes of *Methylotuvimicrobium alcaliphilum* 20Z Δ*pfk* and ALE strains grown on methane.

**Additional file 7.** The Illumina NGS workflow for RNA-seq experiment.

**Additional file 8.** Primers used for RT-qPCR experiment.

## Data Availability

All data generated or analysed during this study are included in this published article and its supplementary information files.

## Abbreviations

PPi-PFK: Pyrophosphate-dependent 6-phosphofructokinase
PPi: Inorganic pyrophosphate
EMP: Embden–Meyerhof–Parnas
ED: Entner–Doudoroff
ATP-PFK: ATP-dependent 6-phosphofructokinase
ALE: Adaptive laboratory evolution
GSM: Genome-scale metabolic model
MCMC: Markov Chain Monte Carlo
COG: Clusters of Orthologous Groups
NMS: Nitrate mineral salt
SAM: Sequence alignment map
BAM: Binary alignment map
FPKM: Fragments per kilobase of exon per million fragments
FDR: False discovery rate

## References

[1] Kalyuzhnaya MG, Puri AW, Lidstrom ME (2015). Metabolic engineering in methanotrophic bacteria. Metab Eng.

[2] Lee EY (2019). Methanotrophs: microbiology fundamentals and biotechnological applications.

[3] Clomburg JM, Crumbley AM, Gonzalez R (2017). Industrial biomanufacturing: the future of chemical production. Science..

[4] Hwang IY, Nguyen AD, Nguyen TT, Nguyen LT, Lee OK, Lee EY (2018). Biological conversion of methane to chemicals and fuels: technical challenges and issues. Appl Microbiol Biotechnol.

[5] Nguyen AD, Hwang IY, Chan JY, Lee EY (2016). Reconstruction of methanol and formate metabolic pathway in non-native host for biosynthesis of chemicals and biofuels. Biotechnol Bioprocess Eng.

[6] Kalyuzhnaya MG, Yang S, Rozova ON, Smalley NE, Clubb J, Lamb A, Gowda GN, Raftery D, Fu Y, Bringel F (2013). Highly efficient methane biocatalysis revealed in a methanotrophic bacterium. Nat Commun.

[7] Nguyen AD, Hwang IY, Lee OK, Kim D, Kalyuzhnaya MG, Mariyana R, Hadiyati S, Kim MS, Lee EY (2018). Systematic metabolic engineering of *Methylomicrobium alcaliphilum* 20Z for 2, 3-butanediol production from methane. Metab Eng.

[8] Akberdin IR, Thompson M, Hamilton R, Desai N, Alexander D, Henard CA, Guarnieri MT, Kalyuzhnaya MG (2018). Methane utilization in *Methylomicrobium alcaliphilum* 20ZR: a systems approach. Sci Rep.

[9] Akberdin I, Collins D, Hamilton R, Oshchepkov DY, Shukla A, Nicora C, Nakayaku E, Adkins JN, Kalyuzhanaya MG (2018). Rare earth elements alter redox balance in *Methylomicrobium alcaliphilum* 20ZR. Front Microbiol.

[10] Nguyen AD, Park JY, Hwang IY, Hamilton R, Kalyuzhnaya MG, Kim D, Lee EY (2020). Genome-scale evaluation of core one-carbon metabolism in gammaproteobacterial methanotrophs grown on methane and methanol. Metab Eng.

[11] Khmelenina VN, Rozova ON, Trotsenko YA. Characterization of the recombinant pyrophosphate-dependent 6-phosphofructokinases from *Methylomicrobium alcaliphilum* 20Z and *Methylococcus capsulatus* bath. In: Methods in enzymology, vol 495. Edited by Anonymous Elsevier; 2011. p. 1–14. 10.1016/B978-0-12-386905-0.00001-2 21419911

[12] Beschastnyĭ AP, Rozova ON, Khmelenina VN, Trotsenko I (2008). Activities of 6-phosphofructokinases and inorganic pyrophosphatase in aerobic methylotrophic bacteria. Mikrobiologiia..

[13] Long CP, Gonzalez JE, Sandoval NR, Antoniewicz MR (2016). Characterization of physiological responses to 22 gene knockouts in *Escherichia coli* central carbon metabolism. Metab Eng.

[14] Long CP, Gonzalez JE, Feist AM, Palsson BO, Antoniewicz MR (2017). Dissecting the genetic and metabolic mechanisms of adaptation to the knockout of a major metabolic enzyme in *Escherichia coli*. Proc Natl Acad Sci.

[15] McCloskey D, Xu S, Sandberg T, Brunk E, Hefner Y, Szubin R, Feist AM, Palsson BO (2018). Growth adaptation of gnd and sdhCB *Escherichia coli* deletion strains diverges from a similar initial perturbation of the transcriptome. Front Microbiol.

[16] But SY, Egorova SV, Khmelenina VN, Trotsenko YA (2018). Serine-glyoxylate aminotranferases from methanotrophs using different C1-assimilation pathways. Antonie Van Leeuwenhoek..

[17] Eshinimaev BT, Khmelenina VN, Sakharovskii VG, Suzina NE, Trotsenko YA (2002). Physiological, biochemical, and cytological characteristics of a haloalkalitolerant methanotroph grown on methanol. Microbiology.

[18] Nguyen AD, Kim D, Lee EY (2019). A comparative transcriptome analysis of the novel obligate methanotroph *Methylomonas* sp. DH-1 reveals key differences in transcriptional responses in C1 and secondary metabolite pathways during growth on methane and methanol. BMC Genomics..

[19] Charusanti P, Conrad TM, Knight EM, Venkataraman K, Fong NL, Xie B, Gao Y, Palsson BØ (2010). Genetic basis of growth adaptation of Escherichia *coli* after deletion of pgi, a major metabolic gene. PLoS Genet.

[20] Wytock TP, Fiebig A, Willett JW, Herrou J, Fergin A, Motter AE, Crosson S (2018). Experimental evolution of diverse *Escherichia coli* metabolic mutants identifies genetic loci for convergent adaptation of growth rate. PLoS Genet.

[21] Sah S, Varshney U (2015). Impact of mutating the key residues of a bifunctional 5, 10-methylenetetrahydrofolate dehydrogenase-cyclohydrolase from *Escherichia coli* on its activities. Biochemistry (NY)..

[22] Conrad TM, Frazier M, Joyce AR, Cho B, Knight EM, Lewis NE, Landick R, Palsson BØ (2010). RNA polymerase mutants found through adaptive evolution reprogram *Escherichia coli* for optimal growth in minimal media. Proc. Natl. Acad. Sci..

[23] Slamovits CH, Keeling PJ (2006). Pyruvate-phosphate dikinase of oxymonads and parabasalia and the evolution of pyrophosphate-dependent glycolysis in anaerobic eukaryotes. Eukaryot Cell.

[24] Khmelenina VN, Rozova ON, Akberdin IR, Kalyuzhnaya MG, Trotsenko YA. Pyrophosphate-dependent enzymes in methanotrophs: new findings and views. In: Methane biocatalysis: paving the way to sustainability. Edited by Anonymous Springer; 2018. p. 83–98.

[25] Deng YW, Ro SY, Rosenzweig AC (2018). Structure and function of the lanthanide-dependent methanol dehydrogenase XoxF from the methanotroph *Methylomicrobium buryatense* 5GB1C. J Biol Inorg Chem..

[26] Utrilla J, O’Brien EJ, Chen K, McCloskey D, Cheung J, Wang H, Armenta-Medina D, Feist AM, Palsson BO (2016). Global rebalancing of cellular resources by pleiotropic point mutations illustrates a multi-scale mechanism of adaptive evolution. Cell Syst..

[27] Liu H, Sun Y, Ramos KR, Nisola GM, Valdehuesa KN, Lee WK, Park SJ, Chung WJ (2013). Combination of Entner-Doudoroff pathway with MEP increases isoprene production in engineered *Escherichia coli*. PLoS ONE.

[28] Cheng K, Lee B, Masuda T, Ito T, Ikeda K, Hirayama A, Deng L, Dong J, Shimizu K, Soga T (2014). Global metabolic network reorganization by adaptive mutations allows fast growth of *Escherichia coli* on glycerol. Nat Commun..

[29] Ojala DS, Beck DA, Kalyuzhnaya MG. Genetic systems for moderately halo (alkali) philic bacteria of the genus *Methylomicrobium*. In: Methods in enzymology, vol. 495. Edited by Anonymous Elsevier; 2011. p. 99–118. 10.1016/B978-0-12-386905-0.00007-3 21419917

[30] Gao Y, Yurkovich JT, Seo SW, Kabimoldayev I, Dräger A, Chen K, Sastry AV, Fang X, Mih N, Yang L, Eichner J, Cho B, Kim D, Palsson BO (2018). Systematic discovery of uncharacterized transcription factors in *Escherichia coli* K-12 MG1655. Nucleic Acids Res.

[31] Langmead B, Trapnell C, Pop M, Salzberg SL (2009). Ultrafast and memory-efficient alignment of short DNA sequences to the human genome. Genome Biol.

[32] Trapnell C, Roberts A, Goff L, Pertea G, Kim D, Kelley DR, Pimentel H, Salzberg SL, Rinn JL, Pachter L (2012). Differential gene and transcript expression analysis of RNA-seq experiments with TopHat and Cufflinks. Nat Protoc.

[33] Deatherage DE, Barrick JE. Identification of mutations in laboratory-evolved microbes from next-generation sequencing data using breseq. In: Engineering and analyzing multicellular systems. Edited by Anonymous Springer; 2014. p. 165–188. 10.1007/978-1-4939-0554-6_12 PMC4239701 24838886

[34] Ebrahim A, Lerman JA, Palsson BO, Hyduke DR (2013). COBRApy. Constraints-based reconstruction and analysis for python. BMC Syst Biol.

[35] Kim D, Seo SW, Gao Y, Nam H, Guzman GI, Cho B, Palsson BO (2018). Systems assessment of transcriptional regulation on central carbon metabolism by Cra and CRP. Nucleic Acids Res.

[36] Schellenberger J, Palsson BØ (2009). Use of randomized sampling for analysis of metabolic networks. J Biol Chem.

[37] Henard CA, Smith HK, Guarnieri MT (2017). Phosphoketolase overexpression increases biomass and lipid yield from methane in an obligate methanotrophic biocatalyst. Metab Eng.

