# Supplementary material for: Metabolic role of pyrophosphate-linked phosphofructokinase pfk for C1 assimilation in Methylotuvimicrobium alcaliphilum 20Z
Source: Microb Cell Fact. 2020 Jun 16;19:131. doi: 10.1186/s12934-020-01382-5 (PMC7298851; doi:10.1186/s12934-020-01382-5)
Supplement: Supplementary file 1 — Additional file 1. Additional figures and legends supporting the results described in text. [file 12934_2020_1382_MOESM1_ESM.docx]

**
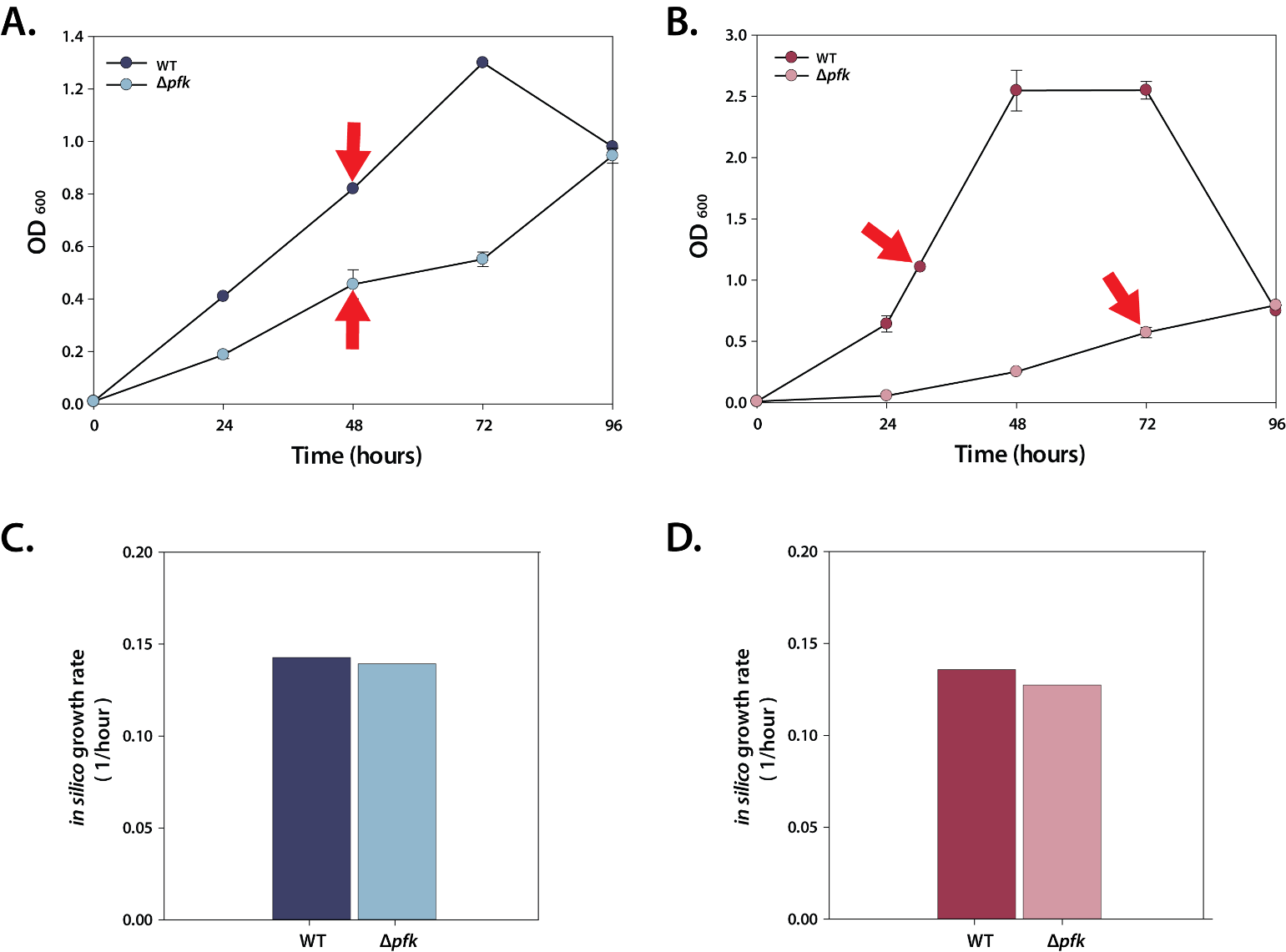
**

**Figure S1**. Comparison growth curve and *in silico* simulation growth rate of *Methylomicrobium alcaliphilum* 20Z wild-type and Δ*pfk* grown on methane (A, C) and methanol (B, D). The initial cell density of each culture was set at OD_600_ = 0.01. The data represent the mean ± SD (n=3). The sampling points for RNA-Seq indicated by red arrows.


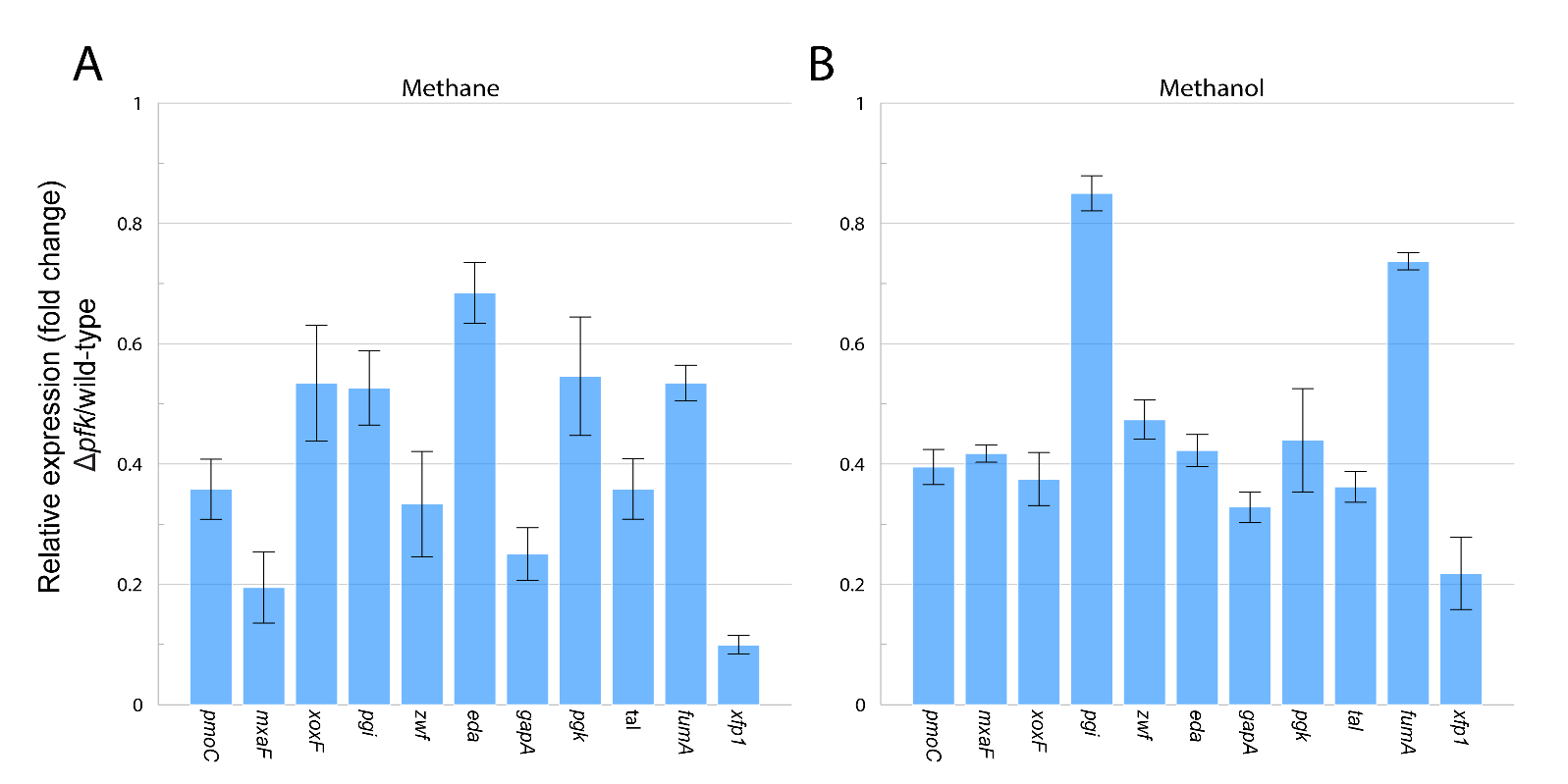


**Figure S2**. RT-qPCR analysis of relative central pathway transcriptions comparing *M. alcaliphilum* 20Z wild-type and Δ*pfk* grown on methane (A) and methanol (B). Abbreviations: *pmoC*: particulate methane monooxygenase; *mxaF*: calcium-dependent methanol dehydrogenase; *xoxF*: lanthanide-dependent methanol dehydrogenase; *pgi*:g-6-phosphate isomerase; *zwf*: glucose-6-phosphate dehydrogenase; *eda*: 2-dehydro-3-deoxyphosphooctonate aldolase; *gapA*: glyceraldehyde 3-phosphate dehydrogenase; *pgk*: phosphoglycerate kinase; *tal*: transaldolase; *fumA*: fumarate hydratase; *xfp1*: D-xylulose 5-phosphate/D-fructose 6-phosphate phosphoketolase. The data represent the mean ± SD (n=2).


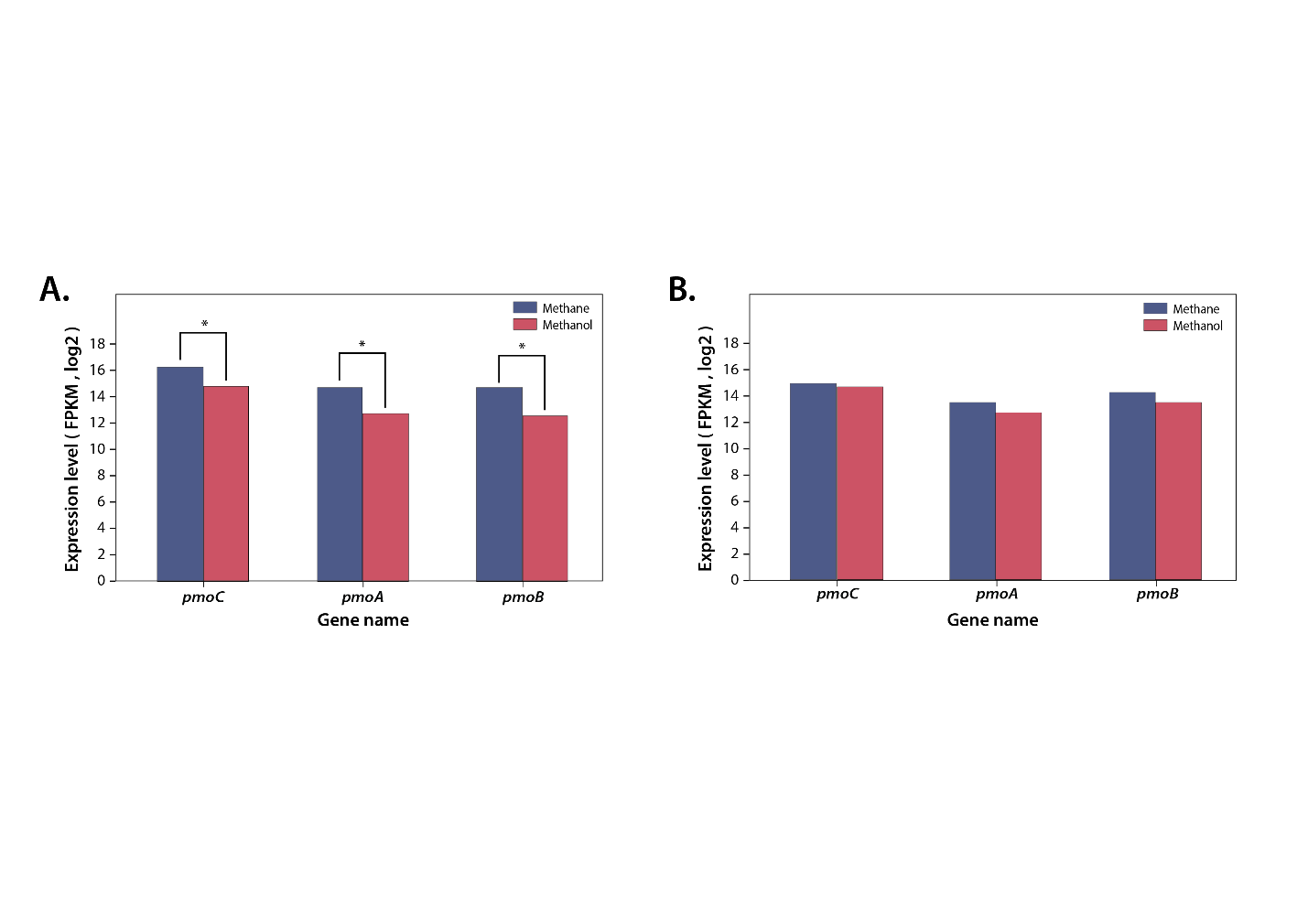


**Figure S3**. Different expression genes involved methane oxidation of *M. alcaliphilum* 20Z wild-type and Δ*pfk* grown on methane and methanol. * Significantly different expression of genes between wild-type and Δ*pf*k (*P* < 0.05).


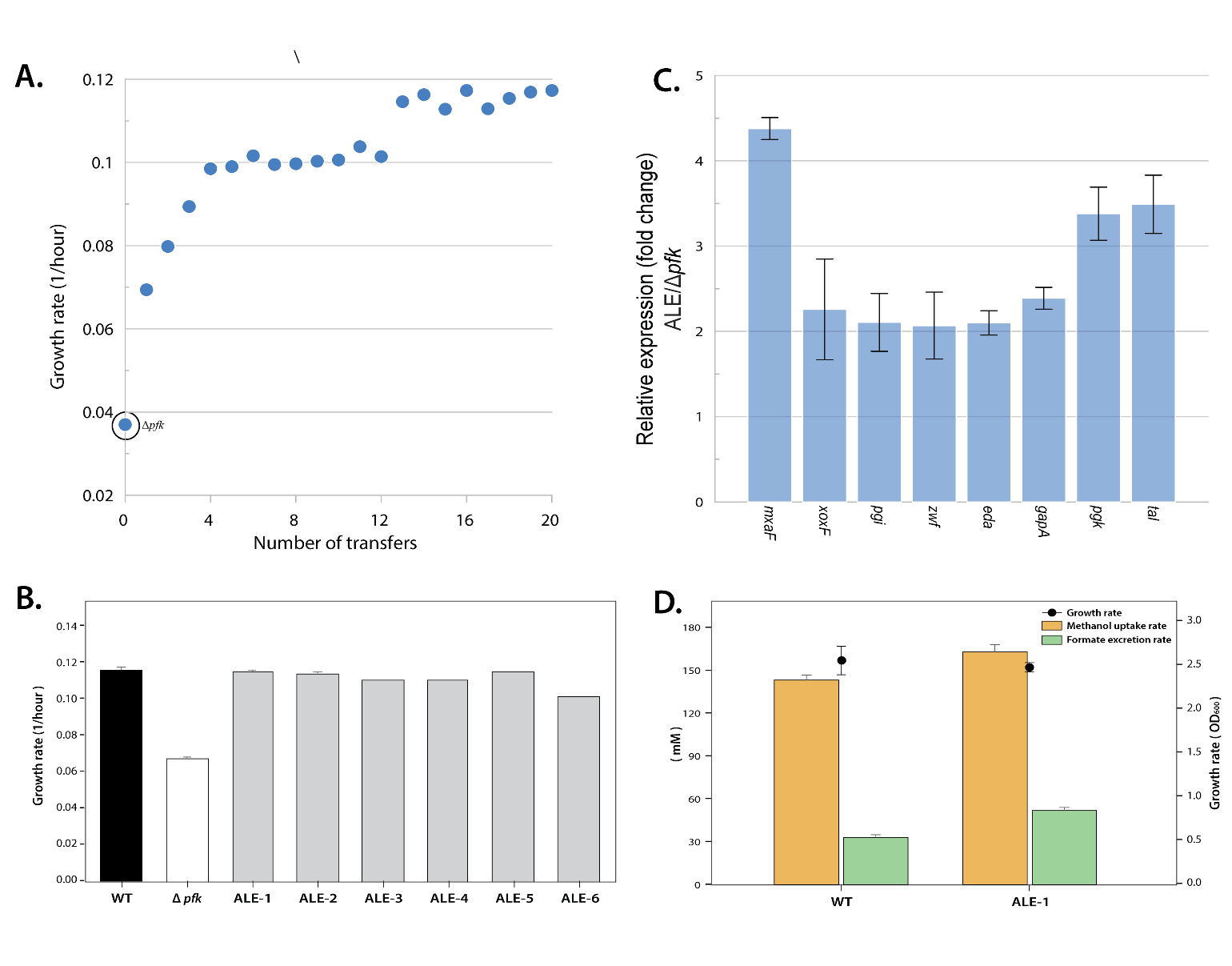


**Figure S4**. Adaptive laboratory evolution trajectory of the initial reference knockout and evolved knockout (A). Growth rate for *M. alcaliphilum* 20Z wild-type, unevolved and evolved strains (B). RT-qPCR analysis of relative central pathway transcriptions comparing ALE strain and Δ*pfk* (C). Methanol uptake rate, formate excretion rate and OD_600_ of *M. alcaliphilum* 20Z wild-type and ALE strain grown on methanol after 48h (D). The initial cell density of each culture was set at OD_600_ = 0.01. The data represent the mean ± SD (n=3). Abbreviations: *mxaF*: calcium-dependent methanol dehydrogenase; *xoxF*: lanthanide-dependent methanol dehydrogenase; *pgi*: glucose-6-phosphate isomerase; *zwf*: glucose-6-phosphate dehydrogenase; *eda*: 2-dehydro-3-deoxyphosphooctonate aldolase; *gapA*: glyceraldehyde 3-phosphate dehydrogenase; *pgk*: phosphoglycerate kinase; *tal*: transaldolase.


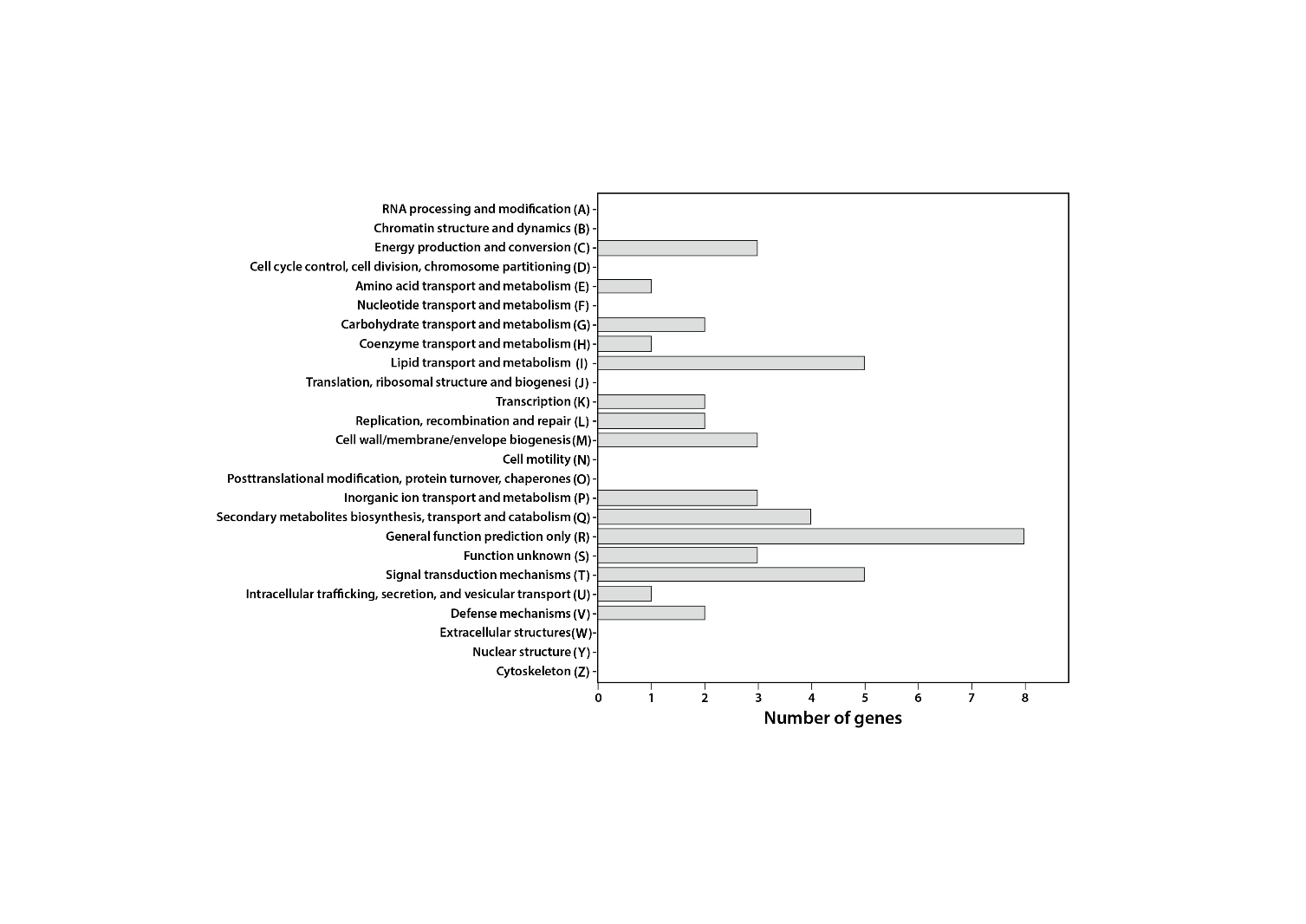


**Figure S5**. Clusters of Orthologous Groups (COGs) analysis of genes appearing mutations in ALE strains.


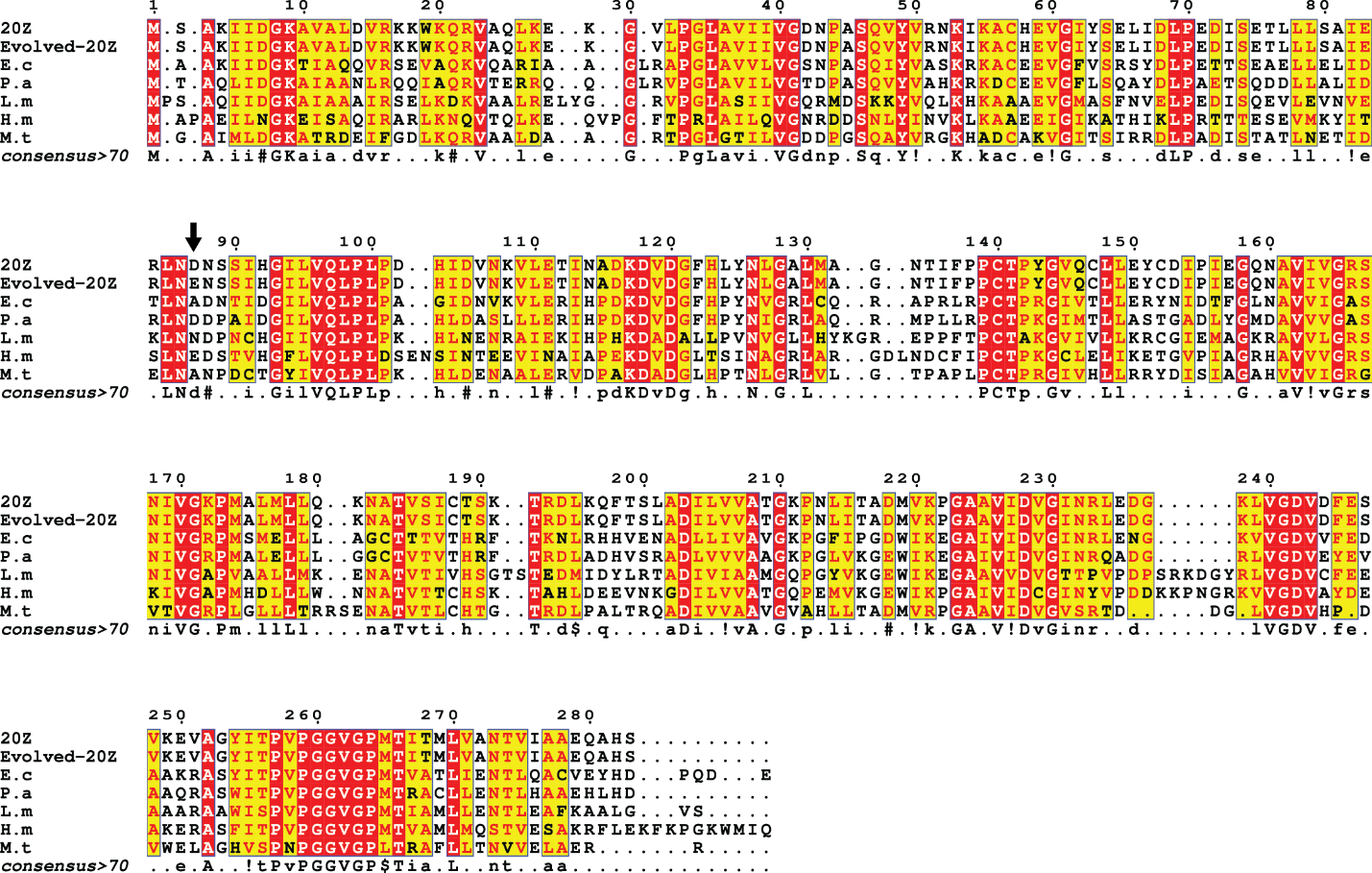


**Figure S6**. Multiple alignments of *folD* sequences highlighting the conserved residues. *folD* sequences from *M. alcaliphilum* 20Z wild-type and evolved strains are aligned with *folD* sequences of *E. coli*, P*. aeruginosa*, *L. major*, *H. sapiens* NMDMC and *M. tuberculosis*.


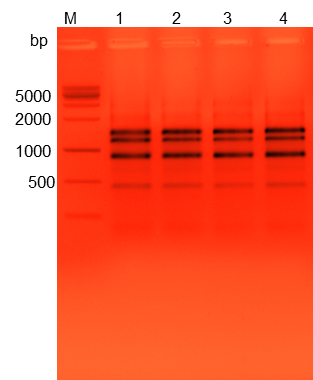


**Figure S7**. Electrophoresis of 1 µg of total RNA samples extracted from *M. alcaliphilum* 20Z wild-type (lane 1, 2) and Δ*pfk* (lane 3, 4) in 1.3% agarose gel and stained with Ethidium bromide.
