# Supplementary material for: Metabolic role of pyrophosphate-linked phosphofructokinase pfk for C1 assimilation in Methylotuvimicrobium alcaliphilum 20Z
Source: Microb Cell Fact. 2020 Jun 16;19:131. doi: 10.1186/s12934-020-01382-5 (PMC7298851; doi:10.1186/s12934-020-01382-5)
Supplement: Supplementary file 7 — Additional file 7. The Illumina NGS workflow for RNA-seq experiment. [file 12934_2020_1382_MOESM7_ESM.docx]

**The Illumina NGS workflow for RNA-seq experiment**

1. **Sample Preparation**

For library construction, RNA is extracted from a sample. After performing quality control (QC), qualified samples proceed to library construction.

1. **Library Construction**

The sequencing library is prepared by random fragmentation of cDNA sample, followed by 5' and 3' adapter ligation. Alternatively, "tagmentation" combines the fragmentation and ligation reactions into a single step that greatly increases the efficiency of the library preparation process. Adapter-ligated fragments are then PCR amplified and gel purified.

1. **Sequencing**

For cluster generation, the library is loaded into a flow cell where fragments are captured on a lawn of surface-bound oligos complementary to the library adapters. Each fragment is then amplified into distinct, clonal clusters through bridge amplification. When cluster generation is complete, the templates are ready for sequencing.

Illumina SBS technology utilizes a proprietary reversible terminator-based method that detects single bases as they are incorporated into DNA template strands. As all 4 reversible, terminator-bound dNTPs are persent during each sequencing cycle, natural competition minimizes incorporation bias and greatly reduces raw error rates compared to other technologies. The result is highly accurate base-by-base sequencing that virtually eliminates sequence-context-specific errors, even within repetitive sequence regions and homopolymers.

1. **Raw data**

Sequencing data is converted into raw data for the analysis. The Illumina sequencer generates raw images utilizing sequencing control software for system control and base calling through an integrated primary analysis software called RTA (Real Time Analysis). The BCL (base calls) binary is converted into FASTQ utilizing illumina package bcl2fastq. Adapters are not trimmed away from the reads
